# Does asthma pay-for-performance program really improve the quality of asthma care: a nationwide retrospective cohort analysis in Taiwan

**DOI:** 10.1186/s12890-025-03673-2

**Published:** 2025-04-25

**Authors:** Pin-Kuei Fu, Tsung-Hsien Yu

**Affiliations:** 1 Department of Medical Research, Taichung Veterans General Hospital, Taichung, Taiwan; 2 Department of Post-Baccalaureate Medicine, College of Medicine, National Chung Hsing University, Taichung, Taiwan; 3 Department of Health Care Management, National Taipei University of Nursing and Health Sciences, No.365, Ming-te Road, Peitou District, Taipei, Taiwan

**Keywords:** Asthma, Pay-for-performance, Health utilization, Medication

## Abstract

**Background:**

Asthma is a prevalent noncommunicable disease worldwide, imposing significant burdens and diminishing the quality of life for those affected. Pay-for-Performance (P4P) programs are reimbursement models that offer incentives to healthcare providers based on their performance metrics. While P4P initiatives have been implemented across various medical conditions, their specific impact on asthma care remains uncertain. This study aims to compare the characteristics and quality of asthma care between patients enrolled in the P4P program and those who are not. Additionally, we will examine trends in these characteristics and care quality over time.

**Methods:**

This study utilized a multiple cross-sectional design to analyze asthma patients diagnosed in 2010 and 2019, drawing data from Taiwan’s National Health Insurance claims database. We collected information on demographic characteristics, P4P program enrollment, medication usage, healthcare service utilization, and attributes of both patients and their primary treatment hospitals. To address the study objectives, we employed logistic regression models and applied 1:1 propensity score matching to mitigate selection bias.

**Results:**

A total of 811,177 individuals diagnosed with asthma were identified, comprising 317,669 in 2010 and 493,508 in 2019. Our findings indicate that patients enrolled in the P4P program had higher prescription rates for inhaled corticosteroids (ICS) and experienced lower rates of hospital admissions and emergency department visits for acute asthma exacerbations compared to non-enrolled patients. We also observed that demographic characteristics influenced P4P enrollment, with these impacts evolving over time. Furthermore, the effects of the P4P program varied across different levels of hospital accreditation.

**Conclusion:**

This study demonstrates that the P4P program positively influences the quality of asthma care. However, variations between P4P and non-P4P enrollers persist and have widened over time. Health authorities should address these disparities to ensure equitable care for all asthma patients.

**Clinical trial number:**

Protocol #202203101RINC.

**Supplementary Information:**

The online version contains supplementary material available at 10.1186/s12890-025-03673-2.

## Introduction

Asthma is a chronic, heterogeneous disease that affects the lower respiratory tract. It is characterized by persistent inflammation and airway hyper-reactivity. This condition leads to symptoms such as coughing, wheezing, breathing difficulty, and chest tightness [1]. According to the 2024 *Global Strategy for Asthma Management and Prevention*, approximately 262 million people worldwide suffer from asthma, with about 1,000 deaths occurring each day due to the condition. The global prevalence is estimated at 9.1% in children, 11.0% in adolescents, and 6.6% in adults [2]. Asthma has a significant impact on health care, contributing to premature death, reduced quality of life, and productivity losses. It ranks 24th among the leading causes of years lived with disability and 34th among the major contributors to disease burden, as measured by disability-adjusted life years. This condition accounts for one-fifth of the total disability-adjusted life years attributed to chronic respiratory diseases [3]. Although asthma incidence and prevalence continue to rise and the disease cannot be cured, it can be controlled, leading to reduced mortality and improved outcomes when managed with regular use of inhaled corticosteroids (ICS) and adherence to an asthma care plan [4]. 

Since the Institute of Medicine’s landmark report *Crossing the Quality Chasm* highlighted the need for aligning incentives to improve care, many countries have initiated new payment systems aimed at enhancing care quality [5]. Pay-for-performance (P4P) schemes, a form of value-based payment, offer financial incentives to health care providers based on achieving specific performance targets and improving patient care quality [6]. In theory, P4P schemes provide stronger incentives for improving care quality compared with traditional fee-for-service models.

In Taiwan, the National Health Insurance (NHI) program introduced P4P programs for five diseases (cervical cancer, diabetes, asthma, breast cancer, and tuberculosis) at the end of 2001 [5, 6]. As of 2024, nine P4P programs are ongoing and reimbursed by the NHI, covering diabetes, early chronic kidney disease, asthma, breast cancer, schizophrenia, hepatitis B, hepatitis C, chronic obstructive pulmonary disease, and pre–end-stage renal disease. Several studies have evaluated the effectiveness of these P4P programs in Taiwan, particularly for diabetes [7, 8], breast cancer [9], chronic obstructive pulmonary disease [10], and pre–end-stage renal disease [11]. Most found that the programs improved care outcomes, though some noted unintended consequences.

The asthma P4P program in Taiwan has been introduced since 2001. Hospitals or clinics can voluntarily participate in this program if they have physicians specializing in Internal Medicine, Pediatrics, Family Medicine, or Otolaryngology, provided that these physicians have completed training in asthma care. Physicians at participating hospitals or clinics have the freedom to recruit patients for the program, and patients have the option to decide whether they would like to enroll.

Asthma was one of the first diseases included in Taiwan’s Pay-for-Performance program; however, research on the asthma P4P was rare. The purpose of this study is to explore who is more likely to be recruited into the P4P program and to assess the effects of P4P on asthma care. Additionally, we also aim to examine whether the enrollment patterns and quality of asthma care differ over time between patients enrolled in the P4P program and those who are not.

## Methods

### Study design

We conducted a nationwide retrospective multiple cross-sectional study. To achieve the study’s objectives, we decided to observe trends in characteristics and care outcomes between the P4P and non-P4P groups over a 10-year period. However, due to the COVID-19 pandemic in Taiwan in 2021 and to account for data availability, we selected 2019 as the second observation year to minimize the impact of COVID-19, therefore, 2010 was selected as the first observation year.

### Data source

For achieving the study purpose, we used Taiwan’s NHI claims data between 2010 and 2011 and between 2019 and 2020 to retrieve information on the demographic characteristics of people with asthma, enrollment status of the asthma P4P program, characteristics of the main facility for asthma care, and the quality of asthma care.

### Inclusion and exclusion criteria

**Fig. 1:**
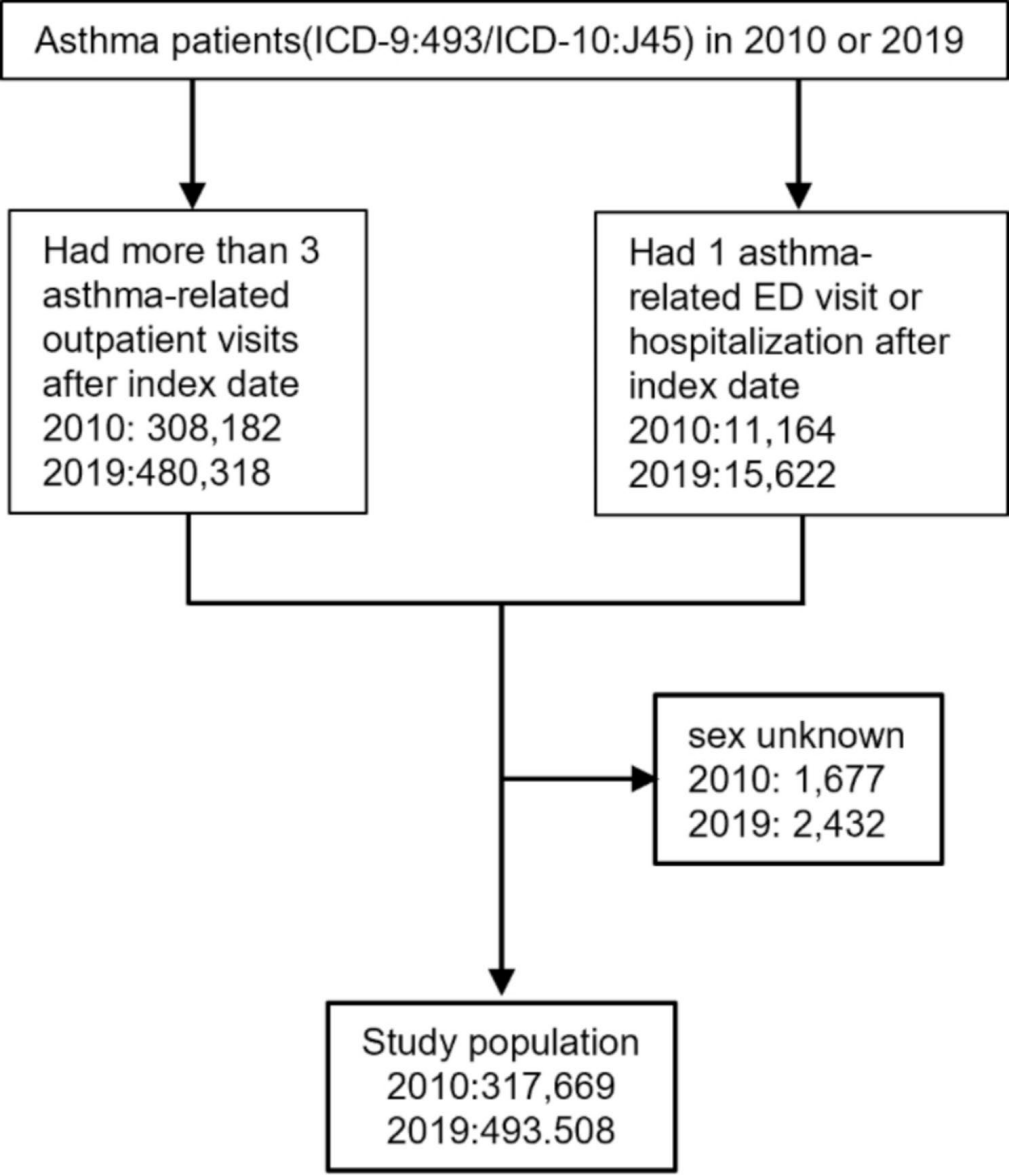
Study population selection. Abbreviation: ED, emergency department

The inclusion criteria for the study were people who (1) were diagnosed with asthma (primary *International Classification of Diseases*,* Ninth Revision* [ICD-9] code 493 or primary *International Classification of Diseases*,* Tenth Revision* [ICD-10] code J45) in 2010 and 2019, and (2) had more than three outpatient visits or at least one emergency department visit or one record of hospitalization within three months after the first asthma diagnosis (index date). Those missing sex data were excluded from the study. Figure 1 outlines the selection process for the study population.

### Variables of interest

#### P4P enrollment

Patients who had more than two consecutive codes of P1612C, P1613C, P1614B, or P1615C in the column of Drug No (in Details of ambulatory care orders) or Order code (Details of inpatient orders) within a year after the index date were P4P program enrollees. Others were non-P4P program enrollees.

#### Quality of asthma care

The rate of Inhaled corticosteroids (ICS) prescription and the rate of Acute asthma exacerbation were selected to represent the quality of asthma care. Inhaled corticosteroids (ICS) are a commonly used treatment for asthma and play a crucial role in managing the condition. The Global Initiative for Asthma recommends their use, making them an important indicator for evaluating the quality of asthma care. For this reason, we have selected ICS use and the occurrence of acute asthma exacerbations as key measures to assess the quality of asthma management. ICS use was defined as the percentage of outpatient visits in which ICS were prescribed. Acute asthma exacerbation was defined as an emergency department visit and admission (primary diagnosis code ICD-9 493 or ICD-10 J45).

### Patient’s demographic characteristics

The demographic characteristics of the patient encompass several key factors, including age, sex, Charlson Comorbidity Index (CCI) score, urbanization level of residence, presence of rare and severe diseases, income status, and the accreditation level of the primary treatment facility. Patient age is categorized into the following groups: under 6 years, 6 to 18 years, 18 to 65 years, and over 65 years. The Charlson Comorbidity Index, developed by Mary Charlson and her colleagues, is a weighted index used to predict the risk of death within one year of hospitalization for patients with specific comorbid conditions. The index includes nineteen conditions, each assigned a weight ranging from 1 to 6. In this study, the comorbidity index score is classified as 0 to 1, 2, or 3 and above.

Income status was based on the patient’s classification under the Taiwan NHI program. Categories 1 to 4 include individuals who are employed, such as civil servants, employees, employers, and farmers or fishermen, as well as military personnel. Category 5 is designated for households that fall below the poverty line. Category 6 is for veterans and other individuals. Dependent family members can enroll through the insurance registration organization of their closest blood relative, such as a parent, spouse, or child. Our study utilized the patient classification under the Taiwan National Health Insurance (NHI) program to determine their low-income status.

In relation to the urbanization level of the residential areas of patients, the NHI in Taiwan operates as an occupation-based social insurance. Unfortunately, claims data does not provide information about the insured individuals’ residences. To address this, we identified the townships of the study population based on the clinics they most frequently visited, which served as a representation of their residence locations. Additionally, we classified all townships in Taiwan into urban or rural categories, following the methodology used in our previous research [12]. 

The health care system in Taiwan comprises medical centers, regional hospitals, community hospitals, and clinics. People in Taiwan are free to select a hospital or clinic for medical services. We defined the main facility for asthma care as the health care facility that the patient most frequently visited.

### Statistical methods

All statistical analyses were performed using SAS (version 9.4, SAS Institute Inc., Cary, NC, USA). In statistical testing, a two-sided *P* value ≤ 0.05 was considered statistically significant. The distributional properties of continuous variables were expressed by mean ± standard deviation, and the categorical variables were presented by frequency and percentage. In univariate analysis, potential predictors of P4P enrollment, ICS prescription, and outcome of asthma care were examined using the chi-square test, the two-sample *t* test, or analysis of variance, as appropriate. Multiple logistic regression was used for multivariable analysis.

For avoiding a situation where physicians might selectively invite patients who could help improve their performance in the P4P program [13], we implemented propensity score matching to reduce the impact of selection bias. The logistic regression model was used to calculate the propensity score for each patient that estimated the probability of their enrollment in the P4P program based on their demographic characteristics (including patient’s age, sex, Charlson Comorbidity Index score, urbanization level of residence, rare and severe diseases, and income status) and the accreditation level of main treatment facility of each patient’s main treatment facility. The caliper matching method with 1:1 matching was used between the P4P and non-P4P groups.

## Results

The study population comprised 317,669 and 493,508 people who were diagnosed with asthma in 2010 and 2019, respectively. A descriptive analysis of the study population is presented in Table 1, showing that the demographic characteristics of the study population changed over time. The average age increased from 34.21 (28.17) years in 2010 to 46.35 (28.84) years in 2019, and the sex ratio became more imbalanced. Additionally, there was an increase in comorbidities. The proportions of patients with severe diseases and those with low income remained similar between the two groups. However, the percentage of patients enrolled in the P4P program decreased from 11.93% in 2010 to 8.83% in 2019. Regarding the main treatment facility, most of the population received asthma care in clinics, although this declined from 58.52% in 2010 to 47.27% in 2019.

**Table 1:** Characteristics of the study population and main treatment facilities, 2010 and 2019

	2010	2019	*P* Value
*n*	317,669	493,508	
**Demographic Characteristics**			
Age, mean (SD)	34.21 (28.17)	46.35 (28.84)	< 0.0001
Age, No. (%)			< 0.0001
< 6 years	86,309 (27.17)	88,231 (17.88)	
7–18 years	52,661 (16.58)	45,302 (9.18)	
19–64 years	114,642 (36.09)	189,691 (38.44)	
65 + years	64,057 (20.16)	170,284 (34.50)	
Sex, No. (%)			< 0.0001
Male	164,117 (51.66)	267,643 (54.23)	
Female	153,552 (48.34)	225,865 (45.77)	
CCI, mean (SD)	0.42 (0.89)	1.67(1.30)	< 0.0001
CCI, No. (%)			< 0.0001
0–1	285,915 (90.00)	344,621 (69.83)	
2	18,841 (5.93)	63,720 (12.91)	
3+	12,913 (4.06)	85,167 (17.26)	
Urbanization level of residence, No. (%)			0.0327
Urban	264,628 (83.30)	411,999 (83.48)	
Rural	53,041 (16.70)	81,509 (16.52)	
Rare and severe disease, No. (%)			0.8818
Yes	44,038 (13.86)	68,472 (13.87)	
No	273,631 (86.14)	425,036 (86.13)	
Low-income status, No. (%)			< 0.0001
Yes	6,236 (1.96)	10,569 (2.14)	
No	311,433 (98.04)	482,939 (97.86)	
**Main Treatment Facility’s Characteristics**			
Accreditation level, No. (%)			< 0.0001
Medical center	40,132 (12.63)	73,621 (14.92)	
Regional hospital	57,442 (18.08)	115,662 (23.44)	
Community hospital	34,207 (10.77)	70,928 (14.37)	
Clinic	185,888 (58.52)	233,297 (47.27)	
P4P enrollee, No. (%)			< 0.0001
Yes	37,911 (11.93)	43,600 (8.83)	
No	279,758 (88.07)	449,908 (91.17)	

Abbreviations: CCI, Charlson Comorbidity Index; No., number; P4P, pay for performance

Table 2 compares the differences in demographic and main treatment facility characteristics between the P4P and non-P4P groups for both years. In 2010, patients under 18 years old (64.98%), males (53.41%), those with no or only one comorbidity (98.01%), urban dwellers (87.44%), those without severe or rare illnesses (8.24%), and those not classified as low-income (1.23%) had a higher likelihood of enrolling in P4P programs. The demographic characteristics of the P4P group and the non-P4P group were still different in 2019, however, their compositions were changed. For instance, while the age structure between the two groups remained significantly different, the proportion of individuals over 19 years old in the P4P group increased significantly. In the non-P4P group, the percentage of those over 19 also rose compared to 2010; however, a notable increase was observed in the percentage of individuals over 65 years old, which rose from 14 to 35.91%. A similar trend was observed in gender distribution. In 2010, both the P4P and non-P4P groups had a higher proportion of men, but by 2019, the participation of women in the P4P group surpassed that of men. Our findings also show that P4P-enrolled patients had a significantly higher rate of ICS prescriptions (10.29% vs. 8.74% in 2010; 12.59% vs. 10.14% in 2019). Additionally, the P4P group experienced significantly lower rates for asthma-related ED visits (1.98% vs. 9.68% in 2010; 1.32% vs. 7.36% in 2019) and admission (0.52% vs. 4.96% in 2010; 0.67% vs. 5.88% in 2019).

**Table 2:** Characteristics of P4P and non-P4P enrollees and main treatment facilities, 2010 and 2019

	2010			2019			Difference Between 2010 and 2019	
	P4P Group	Non-P4P Group	*P* Value	P4P Group	Non-P4P Group	*P* Value	*P* Value	
Age, No. (%)			< 0.001			< 0.001	<.0001^a^	
< 6 years	13,283 (35.04)	73,026 (26.10)		11,094 (25.44)	77,137 (17.15)		<.0001^b^	
7–18 years	11,349 (29.94)	41,312 (14.77)		7,412 (17.00)	37,890 (8.42)		<.0001^b^	
19–64 years	9,783 (25.81)	104,859 (37.48)		16,389 (35.79)	173,302 (38.52)		0.3107^b^	
65 + years	3,496 (9.22)	60,561 (21.56)		8,705 (19.97)	161,579 (35.91)		0.0008^b^	
Sex, No. (%)			< 0.001			< 0.001	<.0001^a^	
Male	20,249 (53.41)	143,868 (51.43)		20,936 (48.04)	246,707 (54.83)		< 0.001^b^	
Female	17,662 (46.59)	135,890 (48.57)		22,644 (51.96)	203,201 (45.17)		< 0.001^b^	
CCI, No. (%)			< 0.001			< 0.001	<.0001^a^	
0–1	36,053 (95.10)	249,862 (89.31)		33,472 (76.77)	311,149 (69.16)		<.0001^b^	
2	1,105 (2.91)	17,736 (6.34)		3,940 (9.04)	59,780 (13.29)		0.1089^b^	
3+	753 (1.99)	12,160 (4.35)		6,188 (14.19)	78,979 (17.55)		<.0001^b^	
Urbanization level of residence, No. (%)			< 0.001			< 0.001	0.4205 ^a^	
Urban	33,150 (87.44)	231,478 (82.74)		38,206 (87.63)	373,793 (83.08)		< 0.001^b^	
Rural	4,761 (12.56)	48,280 (17.26)		5,394 (12.37)	76,115 (16.92)		< 0.001^b^	
Rare and severe disease, No. (%)			< 0.001			< 0.001	0.6172 ^a^	
Yes	3,124 (8.24)	40,914 (14.62)		3,635 (8.34)	64,837 (14.41)		< 0.001^b^	
No	34,787 (91.76)	238,844 (85.38)		39,965 (91.66)	385,071 (85.59)		< 0.001^b^	
Low-income, No. (%)			< 0.001			< 0.001	0.0056 ^a^	
Yes	467 (1.23)	5,769 (2.06)		635 (1.46)	9,934 (2.21)		0.0002^b^	
No	37,444 (98.77)	273,989 (97.94)		42,965 (98.54)	439,974 (97.79)		<.0001^b^	
Main treatment facility’s accreditation level, No. (%)			< 0.001			< 0.001	< 0.0001 ^a^	
Medical center	1,844 (4.86)	38,288 (13.69)		4,015 (9.21)	69,606 (15.47)		< 0.001^b^	
Regional hospital	1,260 (3.32)	56,182 (20.08)		5,202 (11.93)	110,457 (24.55)		< 0.001^b^	
Community hospital	465 (1.23)	33,742 (12.06)		1,911 (4.38)	69,017 (15.34)		< 0.001^b^	
Clinic	34,342 (90.59)	151,546 (54.17)		32,469 (74.48)	200,828 (44.64)		< 0.001^b^	
ED visits, No. (%)	749 (1.98)	27,086 (9.68)	< 0.001	574 (1.32)	33,129 (7.36)	< 0.001	< 0.001^c^	< 0.001^d^
Admission, No. (%)	199 (0.52)	13,882 (4.96)	< 0.001	293 (0.67)	26,442 (5.88)	< 0.001	< 0.001^c^	< 0.001^d^
ICS prescription rates, mean(SD)	10.29 (14.33)	8.74 (14.63)	< 0.001	12.59 (14.21)	10.14 (14.64)	< 0.001	< 0.001^c^	< 0.001^d^

Abbreviations: CCI, Charlson Comorbidity Index; ED, emergency department; ICS, inhaled corticosteroids; No., number; P4P, pay for performance

^a^ comparison between 2010 and 2019; ^b^ comparison between 2010 and 2019 under the same characteristics

^c^ comparison between 2010 and 2019 under the same characteristics P4P; ^d^ comparison between 2010 and 2019 under the same characteristics non-P4P

When we analyzed the data based on the same demographic characteristics, we found significant variations in the proportion of participants in pay-for-performance (P4P) programs over the ten-year period. For instance, in 2010, 12.34% of the male population participated in the P4P program, but this percentage dropped significantly to 7.82% by 2019 (*p* < 0.001). In contrast, although there was still a significant difference in women’s participation rates between the two years, the decline was much less pronounced. The proportion of women participating decreased from 11.50 to 10.05% (*p* < 0.001). In terms of the quality of care, we found that both the P4P group and the non-P4P group experienced significant improvements over the past ten years. Specifically, the P4P group showed a greater improvement in the rate of ICS prescriptions and emergency department visits: 122.35% versus 116.01% for ICS prescriptions, and 33.34% compared to 23.97% for ED visits. However, when it comes to admission rates, both groups saw an increase, with the rise in the P4P group being higher than that in the non-P4P group. Despite this, the proportion of admission in the P4P group remained significantly lower than in the non-P4P group.

**Fig. 2:**
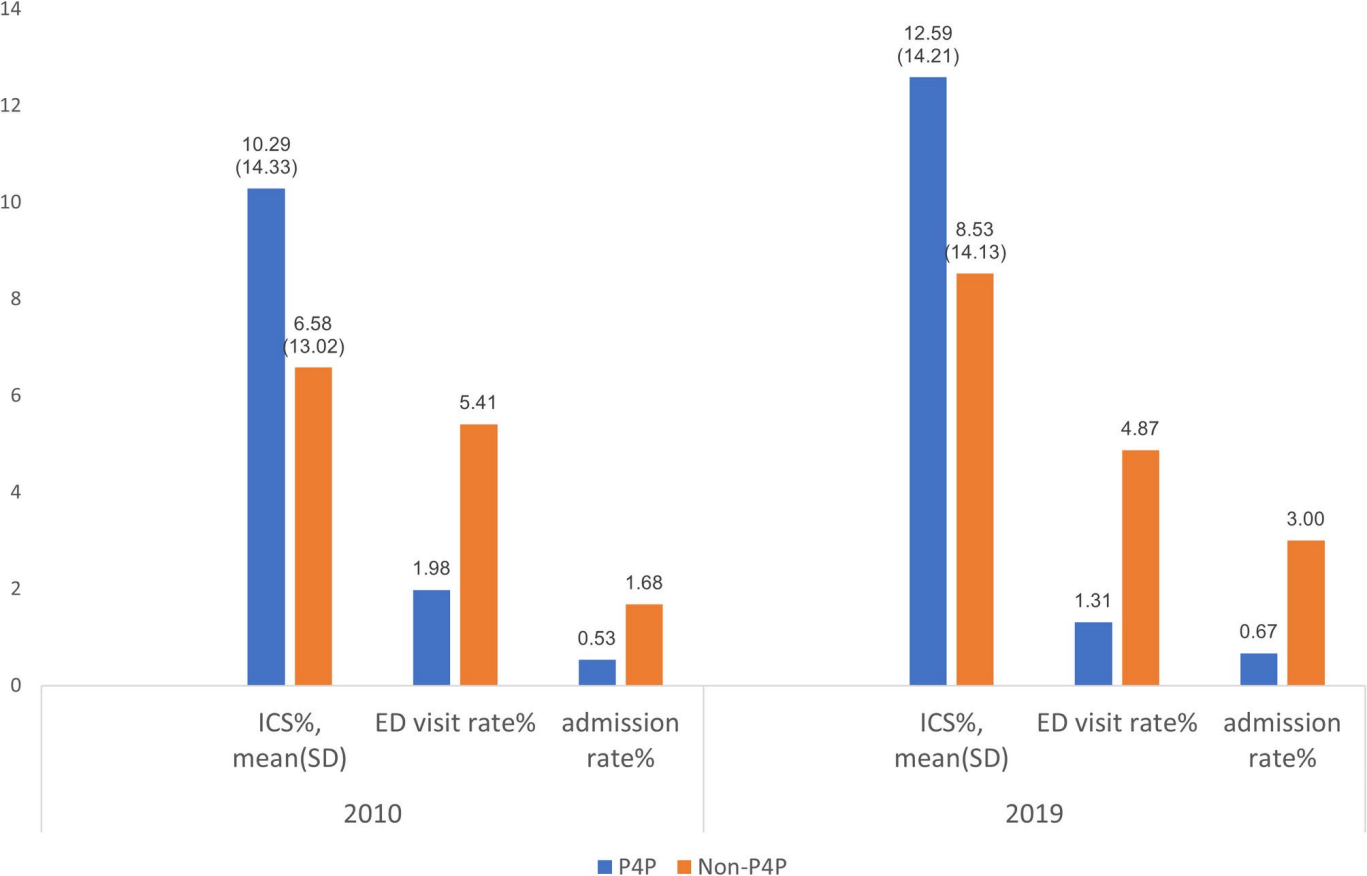
Asthma care outcomes among P4P and non-P4P participants, 2010 and 2019. (after propensity score matching) Note: the ICS prescription rate only represented the ICS prescribed in outpatient visits Abbreviations: ED, emergency department; ICS, inhaled corticosteroids; P4P, pay for performance

Table 3 presents the results of the multivariate analysis. Compared with the P4P group, non-P4P participants were prescribed fewer ICS (β = −3.94% in 2010 and β = −4.03% in 2019). They also had higher odds of ED visit (odds ratio [OR] = 3.02 in 2010 and OR = 3.96 in 2019) and admission (OR = 3.68 in 2010 and OR = 4.85 in 2019), after adjusting for demographic characteristics and accreditation level of main treatment center. To reduce selection bias, we conducted 1:1 propensity score matching. A total of 75,808 (37,094 were from P4P, and 37094 were non-P4P) in 2010 and 87,176 (43,588 vs. 43,588) in 2019 were selected (see Table 4). We present the results in Fig. 2, which show that P4P participants were prescribed more ICS, and had experienced better asthma care outcomes compared with non-P4P participants.

**Table 3:** Results of multivariable analysis

	ICS Prescriptions rate			ED visit				Admission			
	ß	SE	*P* Value	OR	LCL	UCL	*P* Value	OR	LCL	UCL	*P* Value
2010											
P4P enrollment (ref = yes)	−3.94	0.08%	< 0.001	3.02	2.80	3.25	< 0.001	3.68	3.19	4.24	< 0.001
2019											
P4P enrollment (ref = yes)	−4.03	0.07%	< 0.001	3.96	3.64	4.30	< 0.001	4.85	4.32	5.45	< 0.001

Abbreviations: ICS, inhaled corticosteroids; LCL, lower control limit; OR, odds ratio; P4P, pay for performance; SE, standard error; UCL, upper control limit

Note: Data were adjusted based on patients’ demographic characteristics and characteristics of the main facility for asthma care

**Table 4:** Characteristics of P4P and Non-P4P enrollees and main treatment facilities after propensity score matching, 2010 and 2019

	2010			2019		
	P4P (*n* = 37,904)	Non-P4P (*n* = 37,904)	*P value*	P4P (*n* = 43,588)	Non-P4P (*n* = 43,588)	*P value*
Age, No. (%)			1.000			1.000
< 6	13,278(35.03)	13,278(35.03)		11,088(25.44)	11,088(25.44)	
7–18 years	11,347(29.94)	11,347(29.94)		7,408(17.00)	7,408(17.00)	
19–64 years	9,783(25.81)	9,783(25.81)		16,387(37.60)	16,387(37.60)	
65 + years	3,496(9.22)	3,496(9.22)		8,705(19.97)	8,705(19.97)	
Sex, No. (%)			1.000			1.000
Male	20,247(53.42)	20,247(53.42)		20,934(48.03)	20,934(48.03)	
Female	17,657(46.58)	17,657(46.58)		22,654(51.97)	22,654(51.97)	
CCI, N(%)			1.000			1.000
0–1	36,049(95.11)	36,049(95.11)		33,469(76.78)	33,469(76.78)	
2	1,102(2.91)	1,102(2.91)		3,934(9.03)	3,934(9.03)	
3+	753(1.99)	753(1.99)		6,185(14.19)	6,185(14.19)	
Urbanization level of residence, No. (%)			1.000			1.000
Urban	33,143(12.56)	33,143(12.56)		38,199(87.64)	38,199(87.64)	
Rural	4,761(87.44)	4,761(87.44)		5,389(12.36)	5,389(12.36)	
Rare and severe disease, No. (%)			1.000			1.000
Yes	3,117(8.22)	3,117(8.22)		3,629(8.33)	3,629(8.33)	
No	34,787(91.78)	34,787(91.78)		39,959(91.67)	39,959(91.67)	
Low-income, No. (%)			1.000			1.000
Yes	466(1.23)	466(1.23)		627(1.44)	627(1.44)	
No	37,438(98.77)	37,438(98.77)		42,961(98.56)	42,961(98.56)	
Main treatment facility’s accreditation level, No. (%)			1.000			1.000
Medical center	1,844(4.86)	1,844(4.86)		4,014(9.21)	4,014(9.21)	
Regional hospital	1,257(3.32)	1,257(3.32)		5,202(11.93)	5,202(11.93)	
Community hospital	461(1.22)	461(1.22)		1,910(4.38)	1,910(4.38)	
Clinic	34,342(90.6)	34,342(90.6)		32,462(74.47)	32,462(74.47)	

Table 5 compares hospital performance across different accreditation levels. In 2010, the ICS prescription rate was consistently higher among P4P participants across all hospital levels, with medical centers showing the highest prescription rates. Similarly, P4P participants had lower rates of emergency department visits and admission than non-P4P participants, with medical centers showing the best performance. Over a decade, health care use patterns also changed. Among P4P participants, the ED visit and admission rates declined, with community hospitals showing the greatest improvement in ED visits (0.06% in 2010 vs. 0.03% in 2019) and medical centers showing the most significant reduction in admission rates. In terms of ICS prescriptions, P4P participants at regional hospitals saw a 2% increase, whereas those at community hospitals saw a 2% decrease.

**Table 5:** Hospital performance across accreditation levels

	Rate of ED visit	*P* value	Rate of admission	*P* value	ICS	*P* value
2010						
Medical center		< 0.001				0.04
P4P	0.06(0.03)		0.06(0.11)		0.18(0.06)	
Not-P4P	0.15(0.07)		0.07(0.03)		0.14(0.02)	
Regional Hospital		< 0.001		< 0.001		0.0056
P4P	0.09(0.04)		0.05(0.05)		0.15(0.06)	
Not-P4P	0.20(0.12)		0.14(0.12)		0.11(0.04)	
Community Hospital						< 0.001
P4P	0.28(0.36)		0.22(0.38)		0.16(0.12)	
Not-P4P	0.19(0.16)		0.17(0.16)		0.06(0.06)	
Clinics		< 0.001		**		< 0.001
P4P	0.07(0.12)		0.06(0.16)		0.12(0.10)	
Not-P4P	0.19(0.23)		0.13(0.21)		0.06(0.09)	
2019						
Medical center		< 0.001		< 0.001		0.0038
P4P	0.03(0.01)		0.02(0.01)		0.18(0.04)	
Not-p4p	0.09(0.04)		0.07(0.04)		0.13(0.02)	
Regional Hospital		< 0.001		< 0.001		< 0.001
P4P	0.05(0.07)		0.04(0.05)		0.17(0.05)	
Not-p4p	0.13(0.06)		0.12(0.05)		0.12(0.04)	
Community Hospital		< 0.001				< 0.001
P4P	0.04(0.03)		0.14(0.25)		0.14(0.08)	
Not-p4p	0.13(0.14)		0.18(0.21)		0.08(0.06)	
Clinics		< 0.001		< 0.001		< 0.001
P4P	0.04(0.08)		0.04(0.07)		0.12(0.09)	
Not-p4p	0.19(0.24)		0.16(0.23)		0.08(0.09)	

Abbreviations: ED, emergency department; ICS, inhaled corticosteroids; P4P, pay for performance

## Discussion

In this study, we found that demographic characteristics varied between the P4P and non-P4P groups, with these differences changing over time. The P4P group also had a higher rate of ICS prescriptions and fewer emergency department visits and admissions compared to the non-P4P group, with these differences widening over time.

While the findings are promising, it’s unclear if the P4P scheme actually improved asthma care, as existing studies yield inconclusive results influenced by the P4P schemes’ design and incentives. Additionally, most research uses cross-sectional designs, which capture a single time point and cannot establish causality. The ideal study design to assess the effects of the P4P program is a before-and-after study, preferably randomized and controlled. However, the best time to conduct such a study would be at the onset of the program’s implementation [14, 15]. Since the P4P program in Taiwan has been in place since 2001, it presents a challenge. In practice, if physicians anticipate that a patient will join the P4P program, they typically invite the patient at the beginning of treatment. It is uncommon for patients to receive an invitation after they have already started treatment. As a result, the before-and-after study design may not be suitable for this situation. Consequently, we opted for an alternative approach and utilized a multiple cross-sectional study design to observe long-term trends. Similar to an opinion poll during an election, this long-term observation allows us to derive valuable insights from the results.

Furthermore, some researchers have argued that the observed positive outcomes may simply reflect preexisting trends rather than the true effectiveness of the intervention [16, 17]. Our findings indicate that, overall, with the performance of the P4P group significantly surpassing that of the non-P4P group, and the differences between the two groups widening over time. Even after applying propensity score matching, the findings remained consistent.

Third, Does the P4P scheme contribute to disparities in healthcare? Our findings reveal that while most demographic variables are similar among groups, there are notable differences in age, sex, and comorbidity index. Between 2010 and 2019, significant variations in P4P participation rates were observed based on age, gender, comorbidity, and income. This raises concerns about potential issues like cherry-picking and adverse selection, which could worsen existing healthcare disparities [13, 18, 19]. Although we lack data on asthma severity from the Taiwan National Health Insurance claims database, we can examine demographic patterns for signs of cherry-picking. Asthma requires consistent medication for effective symptom control. Studies show that elderly patients tend to have lower medication adherence [20], while those with multiple comorbidities often adhere better [21, 22]. This may explain the financial motivation to include patients with multiple conditions in P4P programs, but it does not clarify the lower participation rates among older adults. Current evidence does not conclusively prove cherry-picking, but it underscores significant participation differences among demographic groups. To maintain equity, health authorities must address the risks associated with cherry-picking in P4P initiatives.

Fourth, our findings indicated that the proportion of inhaled corticosteroid (ICS) prescriptions increased over a 10-year period, with a higher increase rate in the P4P group compared to the non-P4P group. ICS is essential for asthma management, as recommended by the Global Initiative for Asthma (GINA) guidelines [23]. This suggests that the financial incentives of the P4P program may encourage physicians to adhere more closely to guidelines. However, our study’s ICS prescription rates differ from previous research [24] due to a lack of data on chronic-disease refill prescriptions, as our claims database only captures ICS prescriptions during outpatient visits. In Taiwan, physicians may issue these long-term prescriptions to patients with chronic conditions, allowing them to obtain medications from nearby pharmacies without returning to the clinic, typically covering up to three months of treatment. Future studies should include pharmacy records for a more comprehensive analysis of medication utilization between the two groups.

Lastly, we observed a 3% decline in P4P enrollment rates among asthma patients, despite improvements in clinical outcomes. Notably, by 2019, most asthma patients were receiving care in hospitals rather than clinics, with clinics showing the lowest growth in patient numbers. Additionally, while P4P enrollment rates increased in hospitals, they declined in clinics. These trends may be partly explained by the structure and size of the P4P incentives. By design, P4P programs aim to improve care quality through financial rewards [25]. Taiwan’s asthma P4P program provides bonus payments based on a composite score assigned to each participating hospital/clinic. This score incorporates multiple performance indicators, such as completion of follow-up visits, hospital admission rates, and emergency department visit rates. Hospitals/clinics in the top one-third of performance rankings receive an additional reward of 500 points per patient (with the value of one point typically less than 1 NTD, where 1 NTD ≈ 0.033 USD). However, clinics often lack dedicated staff to manage the administrative workload required for program participation—unlike hospitals. Previous research has shown that the size of financial incentives is a critical factor influencing the success of P4P initiatives [26, 27]. As a result, asthma patients treated in clinics were less likely to be enrolled in the P4P program. Consequently, insufficient incentives may have contributed to the decline in participation in the program.

### Policy recommendations

Based on our study’s findings, we recommend that health authorities prioritize the following actions to enhance the quality and equity of asthma care:

1. Develop a Risk-Adjustment Model: Current evaluations rely on outcome indicators without adjusting for patient severity. A robust risk-adjustment model is essential for fair care outcome assessments, as demonstrated by existing tools for acute asthma.
2. Incorporate Process-Oriented Indicators: Quality evaluation should include process-oriented indicators. The Taiwan National Health Insurance Administration should utilize medication usage rates (e.g., inhaled corticosteroids) as performance measures, which are crucial in assessing care quality.
3. Enhance P4P Program Participation: Participation in the P4P program has declined in clinics. The Taiwan National Health Insurance Administration should investigate this trend and consider increasing financial incentives and reducing administrative burdens to encourage participation.

### Limitations

This nationwide, retrospective, multiple cross-sectional study aimed to examine the characteristics of individuals more likely to join the P4P program and its impact on asthma care. It also explored differences in enrollment patterns and quality of care over time for patients in the P4P program versus those not enrolled. However, several limitations should be noted:

1. The cross-sectional design limits the ability to establish causal relationships.
2. Lack of asthma severity data in the Taiwan National Health Insurance (NHI) claims restricts proper risk adjustment and may indicate selective enrollment.
3. Potential underestimation of inhaled corticosteroid (ICS) prescriptions due to the absence of pharmacy dispensing records.
4. Unmeasured factors like patient and physician preferences impacting P4P enrollment were not considered, highlighting the need for qualitative research to explore these subjective elements further.

## Conclusion

This study found that the implementation of the P4P program positively impacted the quality of asthma care. However, variations in the quality of asthma care existed and have widened over time. Addressing and reducing these differences among demographic characteristics should be the focus of future efforts.

## Electronic supplementary material

Below is the link to the electronic supplementary material.

Supplementary Material 1

## Data Availability

Data cannot be publicly shared because of the restrictions of database administration. The data underlying the results presented in the study are owned and managed by the Department of Statistics, Ministry of Health and Welfare. Data application needs to be approved by the Center for Health and Welfare Data Science (https://dep.mohw.gov.tw/DOS/cp-5119-59201-113.html).
