# Supplementary material for: Does asthma pay-for-performance program really improve the quality of asthma care: a nationwide retrospective cohort analysis in Taiwan
Source: BMC Pulm Med. 2025 Apr 25;25:199. doi: 10.1186/s12890-025-03673-2 (PMC12023352; doi:10.1186/s12890-025-03673-2)
Supplement: Supplementary file 1 — Supplementary Material 1 [file 12890_2025_3673_MOESM1_ESM.docx]

Suppl_1 the results of multivariate analysis:2010

|  | ICS rate | | | ED visit | | | | Hospitalization | | | |
| --- | --- | --- | --- | --- | --- | --- | --- | --- | --- | --- | --- |
|  | beta | se | sig | OR | LCL | UCL | sig | OR | LCL | UCL | sig |
| P4P  (ref=yes) | -0.0394 | 0.0008 | *** | 3.02 | 2.80 | 3.25 | *** | 3.68 | 3.19 | 4.24 | *** |
| Age  (ref=<6) |  |  |  |  |  |  |  |  |  |  |  |
| 7-18 | 0.0363 | 0.0008 | *** | 1.13 | 1.09 | 1.18 | *** | 0.72 | 0.68 | 0.78 | *** |
| 19-64 | 0.0726 | 0.0007 | *** | 0.68 | 0.66 | 0.70 | *** | 0.80 | 0.76 | 0.84 | *** |
| 65+ | 0.0367 | 0.0008 | *** | 0.61 | 0.59 | 0.64 | *** | 1.22 | 1.16 | 1.29 | *** |
| Gender  (ref=male) | 0.0006 | 0.0005 |  | 0.88 | 0.86 | 0.90 | *** | 1.35 | 1.30 | 1.39 | *** |
| CCI(ref=0-1) |  |  |  |  |  |  |  |  |  |  |  |
| 2 | -0.0081 | 0.0011 | *** | 1.16 | 1.10 | 1.22 | *** | 1.54 | 1.46 | 1.64 | *** |
| 3+ | -0.0096 | 0.0014 | *** | 1.21 | 1.14 | 1.29 | *** | 1.86 | 1.74 | 1.98 | *** |
| Urbanization level of residence (ref=urban) | -0.0178 | 0.0007 | *** | 1.22 | 1.18 | 1.27 | *** | 1.25 | 1.19 | 1.31 | *** |
| Rare and severe disease  (ref=no) | -0.0065 | 0.0008 | *** | 1.09 | 1.05 | 1.14 | *** | 1.30 | 1.24 | 1.36 | *** |
| Low-income  (ref=no) |  |  |  |  |  |  |  |  |  |  |  |
| Main treatment facility’s accreditation level (ref=medical center) |  |  |  |  |  |  |  |  |  |  |  |
| Regional hospital | -0.0258 | 0.0009 | *** | 1.59 | 1.53 | 1.65 | *** | 1.73 | 1.64 | 1.82 | *** |
| Community hospital | -0.0422 | 0.0012 | *** | 1.39 | 1.32 | 1.46 | *** | 1.98 | 1.86 | 2.11 | *** |
| Clinic | -0.0578 | 0.0013 | *** | 0.40 | 0.37 | 0.42 | *** | 0.27 | 0.25 | 0.30 | *** |

Suppl_2 the results of multivariate analysis:2019

|  | ICS rate | | | ED visit | | | | Hospitalization | | | |
| --- | --- | --- | --- | --- | --- | --- | --- | --- | --- | --- | --- |
|  | beta | se | sig | OR | LCL | UCL | sig | OR | LCL | UCL | sig |
| P4P  (ref=yes) | -0.0403 | 0.0007 | *** | 3.96 | 3.64 | 4.30 | *** | 4.85 | 4.32 | 5.45 | *** |
| Age  (ref=<6) |  |  |  |  |  |  |  |  |  |  |  |
| 7-18 | 0.0424 | 0.0008 | *** | 0.91 | 0.86 | 0.95 | *** | 0.61 | 0.57 | 0.67 | *** |
| 19-64 | 0.0860 | 0.0006 | *** | 0.64 | 0.61 | 0.66 | *** | 0.79 | 0.75 | 0.83 | *** |
| 65+ | 0.0517 | 0.0007 | *** | 0.79 | 0.76 | 0.82 | *** | 1.40 | 1.33 | 1.47 | *** |
| Gender  (ref=male) | 0.0104 | 0.0004 | *** | 0.83 | 0.82 | 0.85 | *** | 0.86 | 0.83 | 0.88 | *** |
| CCI(ref=0-1) |  |  |  |  |  |  |  |  |  |  |  |
| 2 | -0.0116 | 0.0007 | *** | 1.01 | 0.97 | 1.04 |  | 1.35 | 1.30 | 1.40 | *** |
| 3+ | -0.0100 | 0.0006 | *** | 0.97 | 0.94 | 1.00 |  | 1.31 | 1.26 | 1.35 | *** |
| Urbanization level of residence (ref=urban) | -0.0070 | 0.0006 | *** | 1.44 | 1.39 | 1.48 | *** | 1.28 | 1.24 | 1.32 | *** |
| Rare and severe disease  (ref=no) | -0.0042 | 0.0006 | *** | 1.33 | 1.29 | 1.37 | *** | 1.60 | 1.55 | 1.65 | *** |
| Low-income  (ref=no) | -0.0117 | 0.0014 | *** | 1.90 | 1.79 | 2.01 | *** | 2.20 | 2.07 | 2.34 | *** |
| Main treatment facility’s accreditation level (ref=medical center) |  |  |  |  |  |  |  |  |  |  |  |
| Regional hospital | -0.0080 | 0.0007 | *** | 1.48 | 1.43 | 1.53 | *** | 1.65 | 1.59 | 1.72 | *** |
| Community hospital | -0.0218 | 0.0008 | *** | 1.16 | 1.11 | 1.20 | *** | 1.62 | 1.55 | 1.70 | *** |
| Clinic | -0.0440 | 0.0011 | *** | 0.37 | 0.35 | 0.39 | *** | 0.28 | 0.27 | 0.30 | *** |
